# Museomics unveil systematics, diversity and evolution of Australian cycad-pollinating weevils

**DOI:** 10.1098/rspb.2023.1385

**Published:** 2023-10-04

**Authors:** Yun Hsiao, Rolf G. Oberprieler, Andreas Zwick, Yu-Lingzi Zhou, Adam Ślipiński

**Affiliations:** ^1^ Australian National Insect Collection, CSIRO, Canberra, Australian Capital Territory 2601, Australia; ^2^ Division of Ecology and Evolution, Research School of Biology, The Australian National University, Canberra, Australian Capital Territory 2601, Australia; ^3^ Institute of Ecology and Evolutionary Biology, National Taiwan University, Taipei, Taiwan

**Keywords:** co-speciation, cycadales, cycad weevils, divergence dating, phylogeny, pollination

## Abstract

Weevils have been shown to play significant roles in the obligate pollination of Australian cycads. In this study, we apply museomics to produce a first molecular phylogeny estimate of the Australian cycad weevils, allowing an assessment of their monophyly, placement and relationships. Divergence dating suggests that the Australian cycad weevils originated from the Late Oligocene to the Middle Miocene and that the main radiation of the cycad-pollinating groups occurred from the Middle to the Late Miocene, which is congruent with the diversification of the Australian cycads, thus refuting any notion of an ancient ciophilous system in Australia. Taxonomic studies reveal the existence of 19 Australian cycad weevil species and that their associations with their hosts are mostly non-species-specific. Co-speciation analysis shows no extensive co-speciation events having occurred in the ciophilous system of Australian cycads. The distribution pattern suggests that geographical factors, rather than diversifying coevolution, constitute the overriding process shaping the Australian cycad weevil diversity. The synchronous radiation of cycads and weevil pollinators is suggested to be a result of the post-Oligocene diversification common in Australian organisms.

## 1. Introduction

Cycads are palm-like gymnosperms, with large pinnate fronds, representing one of the oldest plant groups due to their Mid-Permian origin. Long thought to be wind-pollinated as most other gymnosperms are [1], it has been recently shown that most cycads are primarily pollinated through brood-site mutualisms with insects, especially with beetles and then mostly with weevils (ciophily) [2–8]. As a ‘reward’ for the pollination ‘service’, the male cones ‘provide’ breeding sites for the pollinators and, by way of the microsporophylls and rachis, an abundant and starch-rich food resource for their larvae. Given the old age of cycads, their obligate insect pollination is sometimes portrayed as a ‘300-Myr-old twosome’, making for a good headline in the media [9,10]. However, recent phylogeny estimates indicate that all extant cycads derive from a global Miocene diversification, *ca* 11–20 Ma [11–14]. Moreover, molecular dating of cycad-pollinating thrips has shown their divergence to have occurred in the Late Miocene rather than being of ancient origin [15], suggesting that extant cycad-insect pollination systems may be much younger than formerly thought. While it is now established that cycads are largely ciophilous (weevil-pollinated), the origin and divergence of their weevil pollinators remain unclear.

**Figure 1.**
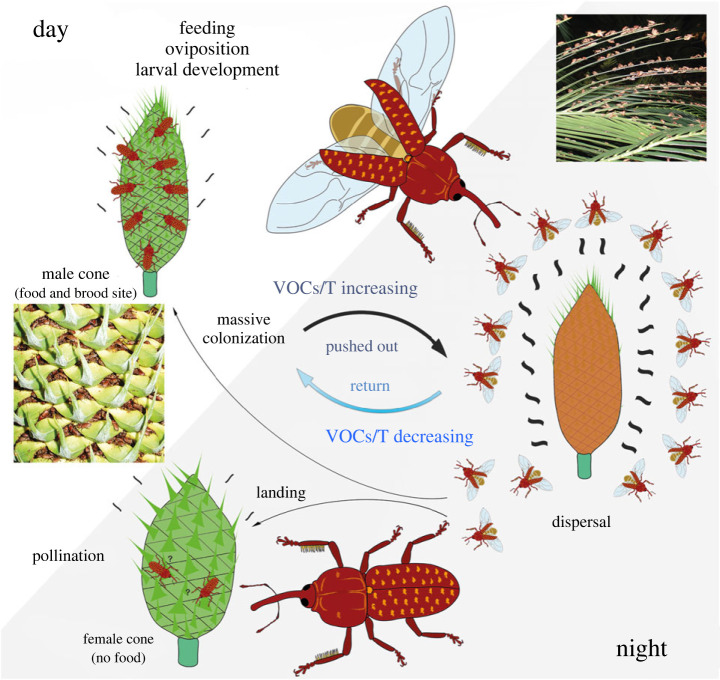
Obligate pollination between *Tranes* weevils and *Macrozamia* cycads. In the daytime, adults aggregate on male cones, where they feed on pollen and oviposit. After sunset, thermogenesis (T) and emissions of volatile organic compounds (VOCs) of cones rapidly increase and adults thus leave the cones and disperse. Weevils return to cones when levels of T and VOCs are lower, and some pollen-laden weevils enter female cones to effect fertilization. Female cones providing neither food nor brood sites may act as a cheating mechanism by mimicking male cones.

With four genera and 85 species (incl. subspecies), Australia is one of the main centres of cycad diversity [16]. Weevils have been shown to play the significant role in the pollination of Australian cycads, even though some beetles families (e.g. Boganiidae) and thrips may also be involved in the pollination of some species [2,6–8,15,17,18]. However, only one Australian cycad–weevil interaction has been studied in detail, that between *Tranes lyterioides* (Pascoe) and *Macrozamia communis* (figure 1) [17,19–21]. Many details of cycad pollination by weevils in Australia remain to be investigated. Obligate associations between weevils and Australian cycads only occur in the weevil family Curculionidae, in two different groups currently classified in the subfamily Molytinae [3–8]. The relationship between the two groups and their monophyly and evolutionary history remain unknown. The *Tranes* group [22] comprises six genera but is morphologically poorly defined, and its tribal placement is controversial, being classified either in the tribe Orthorhinini [23] or in its own tribe [24]. Eleven named species in four genera of the *Tranes* group are truly associated with cycads. Among these, the two species of *Demyrsus* Pascoe and the two of *Siraton* Hustache are trunk borers, their larvae developing in trunks and caudices [25–27], whereas the larvae of *Tranes* Schoenherr and *Miltotranes* Zimmerman develop in male cycad cones and the adults have been confirmed or presumed to be involved in the pollination of their hosts. *Miltotranes* species are tightly associated with *Bowenia* cycads and serve as their pollinators: *M. prosternalis* (Lea) with *B. spectabilis*, *M. subopacus* (Lea) with *B. serrulata* and *M. wilsoni* Hsiao and Oberprieler with the *Bowenia* population in the northern McIlwraith Range [28–30]. *Tranes* comprises four named species and several undescribed species [5,7,17,18,31,32]. *Tranes lyterioides* and its closely related undescribed species have been confirmed to be pollinators of *Macrozamia* [17,19–21,32] and *Lepidozamia peroffskyana* [18]. *Tranes insignipes* Lea breeds in the male cones of *Lepidozamia hopei* and carries the pollen and is thus assumed to pollinate its host [33,34]. *Tranes vigorsii* Boheman, which inhabits the male cones of *Macrozamia riedlei* in Western Australia, probably plays only a minor role in its pollination. If these large weevils do visit receptive female cones, they may perhaps only transfer pollen to the outside and subsequently smaller boganiid beetles and/or thrips may carry the pollen into the cones [17,35]. Similarly, in eastern Australia, the involvement of *Tranes sparsus* Boheman in pollination is uncertain; it breeds in male cones of *Macrozamia* but has not been found to visit female cones [19]. *Cycas* harbours another, as yet undescribed genus of weevils, which appears to be related to the *Tychiodes*-*Nanoplaxes* group that is associated with Asian *Cycas* [36,37]. It apparently pollinates its cycad hosts as well [3,5–7,38], but whether it is closely related to the *Tychiodes*-*Nanoplaxes* group remains unknown.

Altogether, despite the evident significance of weevils in cycad pollination in Australia, the exact nature, diversity and origin of these pollination systems are still unclear. Our study conducts a comprehensive molecular phylogenetic analysis of Australian cycad weevils to investigate their monophyly, systematic position and relationships, combined with divergence dating to estimate their origins. Their species diversity was evaluated based on morphological and molecular differentiation, enabling also clarification of their host specificity. A co-speciation analysis was performed to explore potential co-diversification patterns in the Australian cycad–weevil pollination systems.

Given the unavailability of fresh material of some focal groups and relatives (e.g. the potentially extinct *Howeotranes insularis* (Pascoe)) and challenges experienced in collecting fresh specimens, such as the short cycad coning season and restrictions caused by the extensive 2018–2019 Australian bushfire season and the subsequent COVID-19 pandemic, historical museum-based research formed the core of this project. We used a collection genomics (i.e. ‘museomics’) approach involving low-cost low-coverage genome sequencing to obtain DNA data from dry preserved specimens [39].

## 2. Material and methods

### (a) Species identification

Morphological analysis follows our recent studies [26,28,40]. In total, 3854 weevil specimens collected from 41 Australian cycad species (incl. subspecies) were examined, and at least one pair of specimens from each locality and host species was dissected and compared. Only taxa distinctly supported by clear external and genital characters and by phylogenetic inference were treated as recognizable species. Other weevil species were identified by comparison with authoritatively identified specimens in the Australian National Insect Collection, CSIRO, Australia.

### (b) Taxon sampling and preparation

In total, 120 weevil specimens were selected for whole-genome shotgun sequencing (electronic supplementary material, appendix S1 for sample list). With 79 weevil specimens collected from 22 Australian cycad species, we included nearly all the named and undescribed species of Australian cycad weevils except *Demyrsus digmon* Hsiao and Oberprieler, for which we failed to extract DNA from the only available, old specimens, and a broad coverage of host associations. Despite the uncertain systematic position of the Australian cycad weevils, previous phylogenetic studies supported their placement in the ‘CCCMS clade’ of Curculionidae [41–43]. Consequently, we sequenced additional 41 specimens and downloaded 27 published mitogenomes from NCBI (electronic supplementary material, appendix S1), focusing on representative tribes and genera of the CCCMS clade, especially of Molytinae, as both Australian cycad weevil groups are currently placed in this subfamily. Our phylogeny was rooted with the ‘CEGH clade’ of Curculionidae, the indicated sister-group of the CCCMS clade [41–43]. All specimens were identified to generic level and most to species level. The subfamilial and tribal classification used largely follows [44]. Specimens were sourced from the following Australian collections: Australian National Insect Collection, CSIRO; Queensland Department of Primary Industries; Queensland Museum; South Australian Museum. Ethanol-preserved specimens collected during the last 20 years were used whenever possible to ensure better-quality sequences. Approximately 28% of samples were pinned, dry specimens, ranging from 12 to 133 years old. For our focal group, we selected two to three specimens per species from the same collecting event to improve quality control of the data and to encompass variation in diversity at specific level. Thoracic muscles or one to two legs per beetle were detached and used for DNA extraction. With some rare specimens, only the body was opened for DNA extraction at the connecting membrane between thorax and abdomen, after which all body parts were recovered and remounted.

### (c) DNA extraction, library building and sequencing

Total DNA was extracted using the DNeasy 96 Blood and Tissue Kit protocol (Qiagen). Individual Zymo-Spin IIC columns were used when the number of samples was small (less than or equal to 24). DNA quantity was assessed using a Qubit fluorometer (Thermo Fisher), and DNA fragment sizes were checked using a Fragment Analyser (Agilent). Samples with high-molecular-weight DNA were sheared to approximately 200 bp using a Bioruptor Pico (30 s on, 30 s off for 20 cycles). DNA libraries were built for each sample using the QiaSeq FX DNA library kit with dual-indexed adapters (Qiagen), but only at one-third-volume reactions with 9 µl input DNA. A right- and left-side size selection was performed after PCR amplification (using Beckman Coulter AMPure XP beads at 0.6× then 0.35×). Final libraries were quantified and size-assessed on a Fragment Analyser (Agilent) using the High Sensitivity NGS Fragment Kit (DNF-474). The resulting DNA libraries were normalized equimolar, pooled and sequenced using 150 bp paired-end (PE) reads on a NovaSeq S1 flowcell (Illumina) at the Biomolecular Resource Facility, Australian National University (Canberra, Australia).

### (d) Bioinformatic workflow

Most of the computational analyses were performed on the CSIRO compute clusters Pearcey and Petrichor and on Bowen Cloud resources. The demultiplexed reads were deduplicated with FASTUNIQ v. 1.162 [45] and quality- and adapter-trimmed with TRIMMOMATIC v. 0.36 [46]. Reads were quality-checked using FASTQC v. 0.11.8 [47], and overlapping PE reads were merged with BBMerge v. 38.37 [48]. Merged and remaining separate reads were *de novo* assembled with SPAdes v. 3.13.1 [49]. As nuclear DNA sequences are usually degraded in old dry specimens, we only included mitochondrial DNA in the present study. Mitochondrial protein-coding genes (PCGs) were extracted through comparison against the reference gene set with BLAST + v. 2.10.1 [50]. The mitogenome of *Aclees cribratus* Gyllenhal (NC_051548.1) was selected as reference due to its close relationship with our focal group. To obtain the most complete dataset, we applied an additional, independent workflow using the MitoZ v. 2.3 pipeline [51] to obtain PCGs. The trimmed reads were assembled and annotated on the Pawsey supercomputer at the Australian National University. All resulting genes were aligned with MAFFT plugin v. 3.710 [52] implemented in Geneious Prime 2022.1.1 [53] software and then examined in Aliview v. 1.23 [54]. Any outliers that differed from others in having many alignment gaps were regarded as contaminated sequences and removed. The remaining sequences were concatenated using Geneious, and the more complete one was selected if the two assemblies for the same sample were identical. Any suspicious sequences were temporarily kept in the alignment, and a neighbour-joining tree was built in FastTree v. 2.1.11 [55] to check their placement and recognize contaminant sequences. Also, sequences of cross-contaminants were recognized by having sequences identical to one of the other species; putative cross-contaminants were removed. The surviving sequences, comprising 120 partial to complete mitochondrial genomes, were supplemented with 27 published weevil mitogenomes to yield a final dataset.

The total concatenated nucleotide alignment was 11127 bp long, and poorly aligned codons were visually identified and masked in AliView (936 bp), yielding a final alignment of 10191 bp (electronic supplementary material, appendix S2). The masked alignment was also translated to amino acids and degeneracy-recoded using Degen v. 1.4 [56], recoding all synonymous codons with IUPAC ambiguity codes. This resulted in three final alignments used for downstream analyses: the original nucleotide (NT; 10191 bp), the degeneracy-recoded nucleotide (Degen; 10191 bp) and the amino acid (AA; 3397 sites) alignment.

### (e) Phylogenetic analyses

**Figure 2.**
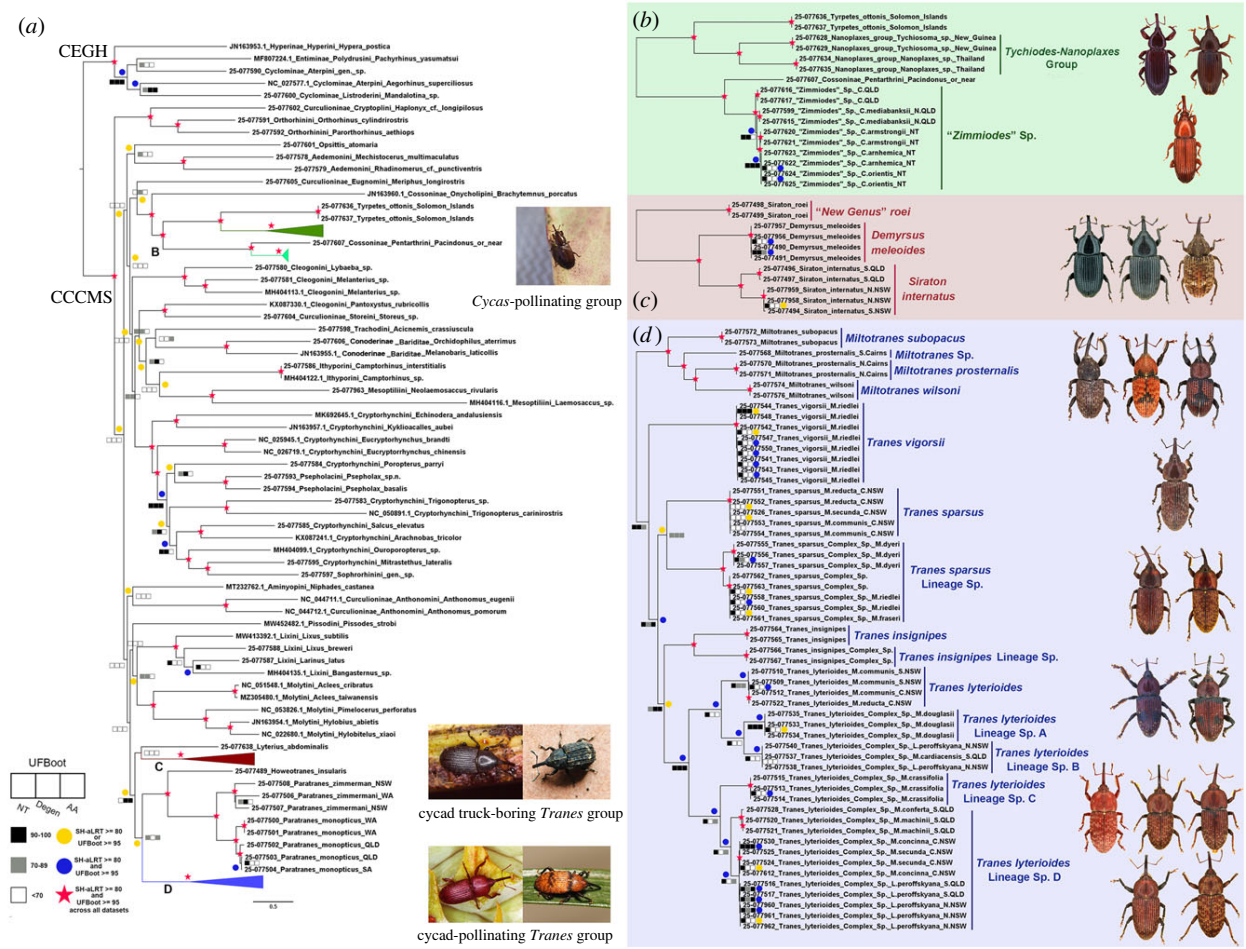
ML phylogeny estimate of Australian cycad weevils based on the NT dataset. Colour shades indicating UFBoot support levels are shown next to each node; yellow circles denoting SH-aRLT ≥ 80% or UFBoot ≥ 95%, blue circles SH-aRLT ≥ 80% and UFBoot ≥ 95% and red asterisks SH-aRLT ≥ 80% and UFBoot ≥ 95% in all datasets. (*a*) Entire tree with clades of Australian cycad weevils represented by coloured triangles (green – *Cycas*-pollinating group (‘*Zimmiodes*’); dark red – cycad trunk-boring weevils of *Tranes* group; blue – cycad-pollinating weevils of *Tranes* group). (*b*) ‘*Zimmiodes*’ and *Tychiodes*-*Nanoplaxes* clade. (*c*) Cycad trunk-boring *Tranes* clade. (*d*) Cycad-pollinating *Tranes* clade.

Phylogenetic relationships were inferred under the maximum-likelihood (ML) optimality criterion in IQ-Tree v .2.1.3 [57,58]. All three datasets were partitioned by gene (13 blocks), with the Degen and NT datasets being additionally analysed partitioned by gene and codon position. The best-fitting partitioning scheme and substitution models were determined by ModelFinder [59,60]. For each dataset and partitioning scheme, at least 200 independent ML tree searches were performed, and the tree with the highest likelihood score was selected as the best-topology tree. Statistical node support was estimated for each tree using Ultrafast Bootstrap (UFBoot) [61] with 10000 replicates and Shimodaira–Hasegawa approximate likelihood ratio test with 10000 replicates (SH-aLRT) [62]. Both support values were mapped for each node onto the respective best-topology tree and are shown combined from all analyses on the preferred topology in figure 2. Nodes with UFBoot ≥ 95 and SH-aLRT ≥ 80 were regarded as strongly supported and those with either UFBoot ≥ 95 or SH-aLRT ≥ 80 as moderately supported. Nodes with UFBoot ≥ 95 across the three analyses and SH-aLRT ≥ 80 were considered to have robust statistical support. Additional Bayesian inference (BI) analyses using MrBayes v. 3.2.7 [63] and BEAST 2 v. 2.6.6 [64] (details in electronic supplementary material, appendix S3) were performed for comparison with the favoured best-topology tree resulting from the ML analyses. Nodes with posterior probability (PP) ≥ 0.95 were considered strongly supported and those with PP = 0.90–0.94 moderately supported. As we included multiple weevil groups in our main analyses, which may have potential effects on the reconstruction of relationships among species in the *Tranes* group containing more species and samples, we ran additional IQ-Tree and MrBayes analyses using smaller taxon combinations containing only the *Tranes* group and seven outgroups to test the stability of the topology. Degen and NT datasets with larger number of characters from three codon positions than AA were herein used. All trees were visualized and edited using iTOL v. 5 [65] (electronic supplementary material, appendix S4).

### (f) Molecular dating analyses

We calculated divergence times using RelTime in MEGA11 [66]. Four fossil records were selected after taxonomic assessment of relevant weevil fossils (electronic supplementary material, appendix S5) and applied for the calibration of nodes in the preferred phylogeny (figure 2). Considering the rapidly evolving nature of mitochondrial genes, we selected the Degen dataset for analysis, as it eliminates all synonymous change and is thus more conservative than the pure NT dataset but still contains more phylogenetic signals than the AA dataset. Local clocks were used for each lineage, with no clock rates merged. The general time reversible model was employed with invariant sites and five discrete rate categories drawn from a γ distribution. The ‘Use all sites’ option was used for all analyses. To evaluate the results from the different types of datasets, we also tested the slowly evolving AA dataset under the LG substitution model, following [43]. As our ML analysis of the AA dataset showed a congruent topology with the phylogeny estimate from the NT dataset, we also tested the Degen and AA datasets under the ML phylogeny based on the AA dataset. Moreover, we performed additional analyses using a relaxed-clock lognormal model implemented in BEAST 2 v. 2.6.6 (details in electronic supplementary material, appendix S6) to test the stability across various divergence-dating methods. The mean heights and 95% highest probability density (95% HPD) were displayed using FigTree v. 1.4.4 [67] (electronic supplementary material, appendix S7).

### (g) Co-speciation analyses

We compared phylogenetic topologies of cycads and weevils to investigate their potential co-speciation patterns using JANE 4 [68]. We focused on *Macrozamia* and the *T. lyterioides* lineage due to its diversity and confirmed role as obligate cycad pollinators. The cycad phylogenetic relationships followed [14], and host associations were based on the label data of examined specimens (electronic supplementary material, appendix S8). We performed analyses with 500 generations and population sizes of 100. We explored how changes in the cost structure associated with co-speciation, duplications of parasites, duplications and host switches, loss of parasites and failure to diverge changed the overall costs of co-speciation between the plants and weevils (table 1). Furthermore, the current *Macrozamia* taxonomy remains poorly resolved, lacking a rigid integrative review of morphological and molecular evidence. The potential oversplitting of host species due to the narrow, ‘morphogeographic’ species concept applied to cycads [69] can be detrimental to such comparative phylogenetic studies. We therefore lumped related host species (based on a recent phylogeny estimate [14]) together and tested two different categories, lumping 39 *Macrozamia* species into nine and four major lineages, and ran additional analyses (electronic supplementary material, appendix S8).

**Table 1.** Results of co-speciation analyses for *Macrozamia* and *Tranes lyterioides* lineage. Changes to the event cost structure (default = 0, 1, 2, 1, 1) suggest that co-speciation events are not major factors in reconciliation between cycad and weevil phylogenies.

type of event						
cost scheme	co-speciation	duplication	duplication and host switch	loss	failure to diverge	overall cost
0, 1, 2, 1, 1	1	2	1	39	22	65
10, 1, 2, 1, 1	0	3	1	40	22	67
0, 10, 10, 1, 1	1	2	1	39	22	91
0, 1, 10, 1, 1	1	3	0	40	22	65
0, 1, 2, 10, 1	1	2	1	39	22	416
0, 1, 2, 1, 10	1	2	1	39	22	263

## 3. Results

### (a) Molecular phylogenetic analyses

The ML analyses of the NT, Degen and AA datasets yielded highly congruent phylogenetic trees of the Australian cycad weevils (figure 2; electronic supplementary material, appendix S4), albeit with variable positions of few lineages of the *Tranes* group and its sister-group. Consistent with morphology, the Australian cycad weevils were recovered as non-monophyletic, separated into three major clades in all analyses. The placement of the *Tranes* group in Molytinae by [22] is confirmed due to its grouping with core molytines, such as the tribes Aminyopini, Lixini, Molytini and Pissodini. In the ML analyses, both the Degen and NT datasets yielded a clade Pissodini + (Lixini + Molytini) as sister-group of the *Tranes* group, but without statistical support, whereas the AA dataset resolved only Lixini as sister-group of the *Tranes* group, with moderate support (SH-aLRT ≥ 80). Our additional BI analyses (using BEAST 2) strongly supported Pissodini + (Lixini + Molytini) in Degen and Lixini + Molytini in NT as sister-group (PP ≥ 0.95; electronic supplementary material, appendix S4), but the AA dataset and MrBayes analyses failed to resolve the relationship or lacked statistical support.

The monophyly of the *Tranes* group was recovered across our various phylogenetic analyses, with moderate statistical support in ML and mostly strong support in BI. Our analyses consistently recovered the southeast Asian *Lyterius abdominalis* (Weber) in the *Tranes* group, but the precise position varied between the main analyses (details in electronic supplementary material, appendix S9). In our main ML analyses, the AA and NT datasets congruently resolved a sister-group relationship between *L. abdominalis* and the cycad trunk borers (i.e. *Demyrsus* and *Siraton*), with moderate statistical support in AA, whereas the Degen dataset moderately supported a close relationship between *L. abdominalis* and the cycad-pollinating group (i.e. *Tranes* and *Miltotranes*). Our main BI analyses were also unable to yield highly consistent results. Nevertheless, all our additional ML and BI analyses of smaller taxon sets supported the relationship between *L. abdominalis* and the cycad trunk borers, with strong support in the MrBayes analysis of the Degen dataset. Additionally, some BI analyses suggested the inclusion of *Niphades* Pascoe (classified in the tribe Aminyopini) in the *Tranes* group, although this placement was also not supported by our analyses of smaller taxon samples. The deeper splits in the *Tranes* group were congruent between the AA and NT analyses, with the cycad trunk borers (including *L. abdominalis*) branching off first and followed by a divergence between the cycad-pollinating group and the non-cycad-associated group (i.e. *Howeotranes* and *Paratranes* Zimmerman), with the node statistically supported by SH-aLRT values in NT. By contrast, analysis of the Degen dataset yielded a close affinity between the cycad trunk borers and the cycad-pollinating groups, but without statistical support. Our additional analyses also predominantly resolved the trunk-boring group as the first split of the *Tranes* group, with strong support.

In the trunk-boring group, *Siraton* was consistently recovered as paraphyletic in all analyses, with the type species, *S. internatus* (Pascoe), forming the sister-group of *Demyrsus*. All our analyses supported the sister-group relationship of *Howeotranes* and *Paratranes* in the non-cycad-associated group, with robust statistical support (details in electronic supplementary material, appendix S9). A monophyletic cycad-pollinating group and the genus *Miltotranes* were recovered across all the analyses, with robust statistical support. The relationship ‘*M. subopacus* + (*M. prosternalis* from south of Cairns + (*M. prosternalis* from north of Cairns + *M. wilsoni*))’ was congruently and robustly supported. *Tranes* was also resolved as a monophylum in all ML analyses, though with weak support. Most BI analyses agreed with ML, with strong support, but a few suggested it to be paraphyletic, with *T. vigorsii* forming the sister-group of a clade *Miltotranes* + the remaining *Tranes*. In *Tranes*, our analyses resolved four major lineages (*T. vigorsii* lineage, *T. sparsus* lineage, *T. insignipes* lineage and *T. lyterioides* lineage). The relationship among these lineages was consistent across the various ML analyses, as *T. vigorsii* lineage + (*T. sparsus* lineage + (*T. insignipes* lineage + *T. lyterioides* lineage)), with moderate to strong support at each node. Additional ML analyses using smaller taxon sampling procured the same results. The BI analyses mostly recovered these relationships as well, with moderate to strong support, but a few resolved different ones, as the *T. insignipes* lineage forming the sister-group of the *T. sparsus* lineage or the *T. sparsus* lineage forming the sister-group of the *T. lyterioides* lineage.

All ML and BI analyses yielded a congruent placement of the *Cycas*-pollinating weevils (i.e. the *Tychiodes*-*Nanoplaxes* group in Asia and the undescribed genus in Australia, provisionally named ‘*Zimmiodes*’) in Cossoninae and also the relationships between them, with robust statistical support across the three datasets. The *Tychiodes*-*Nanoplaxes* group was recovered as sister-group of the Solomon Island genus *Tyrpetes* Heller but ‘*Zimmiodes*’ as sister-group of a species of or near the genus *Pacindonus* Kuschel.

### (b) Divergence dating

**Figure 3.**
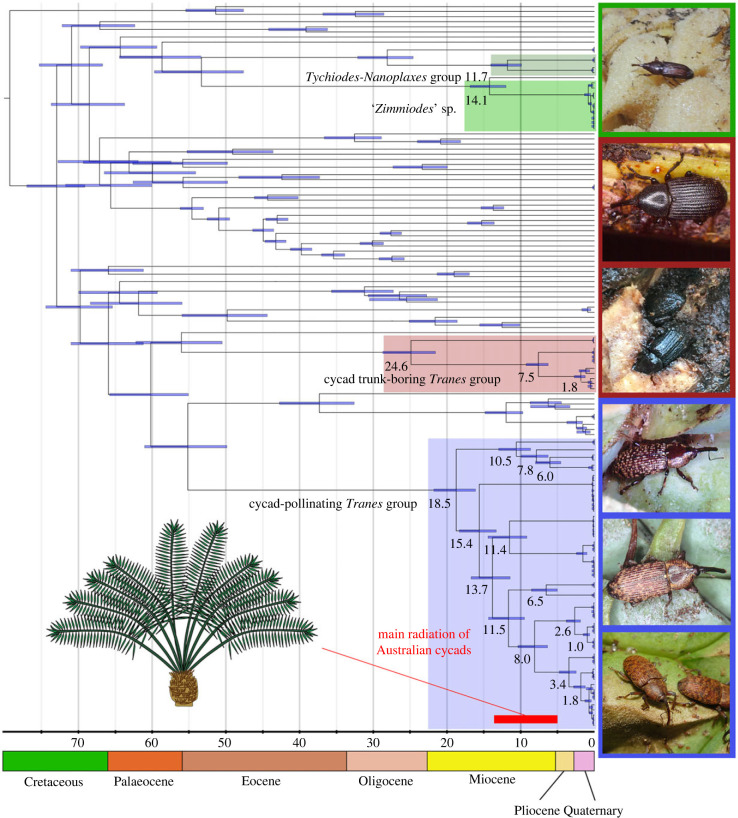
Fossil-calibrated chronogram for Australian cycad weevils showing RelTime estimates of lineage divergence times based on the Degen dataset and preferred ML phylogenetic topology. The outgroups are not shown. Blue bars show 95% confidence intervals of node ages, and the scale bar is in millions of years and geologic time scale is provided. The main divergence of Australian cycads is denoted, revealing a synchronous diversification pattern.

Our dating analyses (figure 3) estimated an Oligocene–Miocene origin of the Australian cycad weevils (*ca* 24.6–14.1 Ma, 95% CI: 28.4–11.9 Ma), with the most recent common ancestor of the cycad trunk borers of the *Tranes* group originating in the Late Oligocene (*ca* 24.6 Ma, 95% CI: 28.4–21.3 Ma), that of the cycad-pollinating taxa of the *Tranes* group in the Early Miocene (*ca* 18.5 Ma, 95% CI: 21.5–15.9 Ma) and that of the *Cycas*-pollinating taxa in the Middle Miocene (*ca* 14.1 Ma, 95% CI: 16.7–11.9 Ma). The major radiation of cycad-pollinating groups is indicated to have occurred from the Middle to the Late Miocene (*ca* 14.1–3.4 Ma). Although one additional RelTime analysis using the AA dataset showed a very wide 95% confidence interval, other tests using RelTime and BEAST 2 produced highly congruent results (electronic supplementary material, appendix S7).

### (c) Co-speciation analysis

Our co-speciation analyses of *Macrozamia* and the *T. lyterioides* lineage revealed no extensive co-speciation events, and changes to the cost structure indicate that co-speciation is not a major factor in reconciliation of cycad and weevil phylogenies (table 1). Additional analyses using two different levels of lumping *Macrozamia* species together yielded consistent results (electronic supplementary material, appendix S8).

## 4. Discussion

### (a) Systematics of Australian cycad weevils

In contrast to Shin *et al.* [43], who recovered a close relationship between *Tranes* and *Vanapa* Pouillaude (of the *Eurhamphus* group of Orthorhinini), none of our analyses revealed an affinity between the *Tranes* and *Orthorhinus* groups, rejecting the inclusion of the *Tranes* group in Orthorhinini [23]. The previous result [43] may be due to insufficient sampling of molytines or to an inappropriate placement of *Vanapa* or of the *Eurhamphus* group in Orthorhinini. The sister-group of the *Tranes* group remains unclear based on our results, which did not yield entirely congruent topologies and strong statistical support for the indicated relationships. Considering the consistency among the various datasets and methods and statistical support values, we regard the clade Pissodini + (Lixini + Molytini) as the most likely sister-group of the *Tranes* group.

Legalov [24] established a separate tribe for the *Tranes* group and compared it with Amalactini, probably because *Tranes* was once classified together with *Amalactus* Schoenherr and *Aorus* Schoenherr. The monophyletic *Tranes* group recovered in our various analyses appears to warrant this classification. However, without any evident synapomorphic characters, it remains poorly defined as a monophylum and thus formally classifiable as a tribe. Furthermore, the southeast Asian *L. abdominalis*, which has a flattened body, separate procoxae and a distinct and complete discrimen on the metaventrite and appears to be associated with *Pandanus*, was recovered in the *Tranes* group, indicating not only that the morphological definition of the whole group requires attention but also that a tribal name for it needs careful deliberation (details in electronic supplementary material, appendix S9). Additionally, *Niphades* was sometimes included in the *Tranes* group in our BI analyses as well. It is evident that the full concept and classificatory position of the *Tranes* group is not yet clear and requires considerable further study, and we therefore refrain from accepting a tribal status and name for the *Tranes* group here. Regarding the major divergences in the *Tranes* group, we consider the relationship ‘cycad trunk-boring group + (cycad-pollinating group + non-cycad-associated group)’ likely to be correct based on our phylogenetic analyses. Furthermore, pertinent morphological characters, namely the absence of sclerolepidia along the metanepisternal sutures, the dorsally open penis with a median groove and the very similar anchor-shape structure of the copulatory sclerite in the endophallus of *Howeotranes*, *Paratranes*, *Miltotranes* and several species of *Tranes* [28,40] indicate a closer relationship of the cycad-pollinating to the non-cycad-associated groups than to the cycad trunk borers.

The relationships among the cycad trunk borers were consistently recovered across all analyses, as *Siraton roei* (Boheman) + (*S. internatus* + *Demyrsus*), suggesting that *S. roei* may be better placed in a different genus, discordant with morphology [26]. The monophyly of the cycad-pollinating group was robustly recovered across all the analyses, supporting the previously postulated close affinity between *Tranes* and *Miltotranes* based on morphological characters [28]. The relationships in *Miltotranes* support the morphological comparison [28], with *M. prosternalis* more closely related to *M. wilsoni* than to *M. subopacus*, but the results also suggest that the *M. prosternalis* populations occurring south of Cairns may represent another, morphologically cryptic but genetically distinct species. Unlike *Miltotranes*, which was strongly supported as a monophyletic group, *Tranes* was weakly resolved as a monophylum in the ML analyses and a few BI analyses suggested it to be paraphyletic, although most strongly supported its monophyly. Considering the topological consistency, statistical support values and the morphological evidence, we consider a monophyletic genus *Tranes* as resolved by most of our analyses as likely to be correct. In *Tranes*, despite the variable positions in a few BI analyses, we favour the relationship ‘*T. vigorsii* lineage + (*T. sparsus* lineage + (*T. insignipes* lineage + *T. lyterioides* lineage))’ resolved by the ML and most of the BI analyses and based on their consistency and statistical support values.

The subfamilial placement of the *Tychiodes*-*Nanoplaxes* group and ‘*Zimmiodes*’ has been controversial. *Tychiodes* Wollaston and *Tychiosoma* Wollaston were originally described in Cossoninae [70,71] but *Nanoplaxes* Heller in ‘Trypetidae’ [72] (Trypetidini of Molytinae) [73] and ‘*Zimmiodes*’ was illustrated as a new genus and species of Cossoninae [74]. Oberprieler [3] recognized the similarity between *Tychiodes* and ‘*Zimmiodes*’ and suggested that they should be placed in Molytinae due to their lack of typical cossonine characters, and he subsequently transferred them to Trypetidini because of their similarity with *Tyrpetes*, which at the time was included in this tribe, though he noted that the tribe was almost certainly a polyphyletic assemblage [5,75]. Lyal [76] later concurred with this assessment, transferring the type genus and its New World relatives to Petalochilini and placing *Tyrpetes* as Molytinae *incertae sedis*. A recent preliminary DNA analysis using a short fragment of 16S rRNA also supported the separation of the *Tychiodes*-*Nanoplaxes* group from Cossoninae [37]. By contrast, our analyses, using a considerably larger DNA dataset, recovered the *Tychiodes*-*Nanoplaxes* group and ‘*Zimmiodes*’ in Cossoninae, consistent with an earlier phylogeny estimate based on a multilocus dataset [42]. Moreover, despite the morphological similarity, a sister-group relationship between the two groups was rejected, with the *Tychiodes*-*Nanoplaxes* group placing as sister-group of *Tyrpetes*—supporting Oberprieler's conclusion [5,75]—but ‘*Zimmiodes*’ as sister-group of a species of *Pacindonus*, belonging to the cossonine tribe Pentarthrini, whose species generally inhabit dead wood. This indicates that these *Cycas*-associated groups are related to Cossoninae rather than Molytinae but that their similarity is likely the result of convergent evolution of inhabiting cones of *Cycas*. It is worth noting that the *Pacindonus* specimen was also collected in the crown of a *Cycas* plant in northern Queensland, although it is doubtful whether the species is exclusively associated with cycads. Furthermore, by their possession of five-segmented funicles, all genera of this clade (including *Tyrpetes*) fit into Pentarthrini, in which they are best placed *pro tempore*, until the concept of this tribe is clarified.

As the most appropriate summary of the results, we deem the ML phylogeny estimate based on the NT dataset (figure 2) to represent the best resolution and most accurate depiction of the phylogenetic relationships and taxon concepts among the Australian cycad-associated weevils.

### (b) Origin and evolution of cycad-association in weevils of Australia

Our dating analyses unveil a synchronous pattern of radiation, the recent species diversification of the Australian cycads (*ca* 13–5 Ma) [14] coinciding with a concomitant diversification in the pollinating weevils in the Middle to Late Miocene. Also congruent with the diversification pattern of cycad-pollinating thrips [15], our results estimate a recent divergence of the Australian cycad-pollinating weevils, revealing not an ancient but a much younger weevil-mediated pollination system in Australia. It also agrees with the notion of a late Tertiary origin for the Australian cycad-pollinating weevils [5]. Our analyses also date the divergence of the *Tychiodes*-*Nanoplaxes* group to the Middle Miocene (*ca* 11.7 Ma, 95% CI: 13.9–9.8 Ma), highlighting the temporal congruence of the evolution of *Cycas*-pollinating weevil groups in Asia and Australia. Together with the indicated divergence time of the cycad-pollinating Allocorynina weevils in the Neotropics [42] and shallow interspecific genetic divergence of the Amorphocerini in Africa [77], it suggests that all extant ciophilous systems of cycads worldwide are of recent origin instead of great antiquity.

### (c) Species diversity, host specificity and speciation

Our morphological studies delimit ten species of *Tranes*, four formerly named and six undescribed (one in the *T. sparsus* lineage, one in the *T. insignipe*s lineage and four in the *T. lyterioides* lineage). The ‘*Zimmiodes*’ cossonines inhabiting the male cones of Australian *Cycas* species are identified as a single species (displaying no significant morphological differences). Except for *D. digmon*, of which we failed to sequence DNA, all the named and undescribed morphospecies are corroborated by our molecular phylogenetic analyses, except for the *M. prosternalis* populations south of Cairns possibly constituting a further, morphologically cryptic species. Due to the sequence of the specimen of *T. lyterioides* lineage sp. B collected from *Macrozamia cardiacensis* being too short, only the NT dataset with the full phylogenetic signal was able to clarify its placement. Altogether, 19 species in six genera, including eight new species, are recognized among the Australian cycad-associated weevil fauna. The new taxa will be described in separate taxonomic papers.

The known host associations (electronic supplementary material, appendix S8) show that the Australian cycad weevils are largely oligophagous, being associated with several cycad species occurring in neighbouring regions, which indicates that coevolution is an unlikely model for their species diversification. The host range of the undescribed sp. D of the *T. lyterioides* lineage even spans the cycad genera *Macrozamia* and *Lepidozamia*, which are not each other's closest relatives (the latter being more closely related to the African *Encephalartos*) [14]. Moreover, while *Tranes* weevils are generally associated with cycad species from the same clade of *Macrozamia*, they can also be associated with species from different clades, enhancing their non-species-specific nature. The large, functional wings of the weevils as well as observations on their flight behaviour in the field indicate that they are fairly strong flyers and can probably disperse quite readily to populations of various cycad species in the same region and thus colonize new hosts.

From an evolutionary point of view, the main interest lies in how this elaborate pollination mutualism shaped the simultaneous diversification in both plants and pollinators. However, despite the temporal correlation of the divergences of the Australian cycads and their pollinating weevils, our co-speciation analyses revealed no extensive co-speciation events and indicated that co-speciation is not a major factor in reconciliation of cycad and weevil phylogenies (table 1). Additional analyses adopting a coarser species concept for cycads resulted in consistent results (electronic supplementary material, appendix S8). Moreover, we could not identify any specific trait of cycads and weevils contributing to a reciprocal evolutionary process of coevolution. Together with the weak hostplant-pollinator specificity, a dominant role of cycad–pollinator interactions in accelerating the diversification of Australian cycad-pollinating weevils appears to be implausible. Instead, the distribution pattern (electronic supplementary material, appendix S10) indicates that allopatric speciation is probably the overriding process shaping the weevil species diversity. The Middle to Late Miocene diversification can likely be a result of the fragmentation of mesic environments by the increasing aridity during this period [78,79]. Such post-Oligocene diversification appears to be a common pattern in Australia, as it can be seen in different organisms (e.g. [80,81]) including cycad trunk-boring weevils, which are unlikely to contribute to plant reproductive success. The hypothesis of co-divergence of cycads and weevils driven by the coevolutionary diversification is, therefore, rejected.

## 5. Conclusion

We reconstructed the first mitogenomic phylogeny estimate of Australian cycad-associated weevils based on museomics. The cycad trunk-boring weevils and cycad-pollinating weevils belong to different clades of the *Tranes* group in Molytinae. The southeast Asian *Lyterius* and the clade comprising *Howeotranes* and the grasstree-associated *Paratranes* are indicated to be sister-groups of the cycad trunk-boring and the cycad-pollinating weevils, respectively. The monophyly of the cycad weevils of the *Tranes* group is confirmed except for a paraphyletic *Siraton*, indicating that *S. roei* may be better placed in a distinct genus. A placement of the Australian *Cycas*-pollinating weevils in Pentarthrini of Cossoninae is recovered, but their close affinity to the Asian cycad weevils (i.e. the *Tychiodes*-*Nanoplaxes* group) is refuted. Dating analyses reveal a Late Oligocene to Middle Miocene origin (*ca* 24.6–14.1 Ma) of the Australian cycad weevils and a main radiation of the cycad-pollinating groups during the Middle to Late Miocene (*ca* 14.1–3.4 Ma), mirroring the age of diversification of the Australian cycads (*ca* 13–5 Ma). The notion of an ancient weevil-mediated pollination of cycads in Australia is thus not supported. Combining these results with morphological studies, we conclude that the Australian cycad weevils comprise 19 species, including eight undescribed ones. Based on their host-association records, the Australian cycad weevils are mostly not species-specific. Our co-speciation analysis also indicates that no extensive co-speciation events shaped their evolutionary histories. The distribution pattern further suggests that geographical factors likely played more significant roles in the speciation of the Australian cycad weevils than host-association factors. The temporal correlation of species radiation in cycads and their weevil pollinators may thus be a result of the post-Oligocene diversification occurring in several Australian organisms.

## Data Availability

The raw reads of all newly generated sequence data are available at the NCBI's SRA. The assembled, cleaned and annotated sequences as used in the alignments have been uploaded to NCBI Genbank. Data available from the Dryad Digital Repository: https://doi.org/10.5061/dryad.v41ns1s2f [82]. Supplementary material is available online [83].
